# Attention-Deficit/Hyperactivity Disorder Patients May Have Undiagnosed Narcolepsy

**DOI:** 10.7759/cureus.8436

**Published:** 2020-06-04

**Authors:** Lukas Wilenius, Markku Partinen

**Affiliations:** 1 Neurology, University of Helsinki, Helsinki, FIN; 2 Helsinki Sleep Clinic, Vitalmed Research Center, Helsinki, FIN; 3 Clinical Neurosciences, University of Helsinki, Helsinki, FIN

**Keywords:** attention deficit hyperactivity disorder (adhd), narcolepsy, serum iron, serum ferritin, adult adhd, excessive daytime sleepiness, cataplexy

## Abstract

Background

Attention-deficit/hyperactivity disorder (ADHD) patients have many comorbidities. Narcoleptic patients have a big prevalence of ADHD (15%-30%). Both groups suffer from similar symptoms and benefit from the same class of medications. As such, narcolepsy could be masked in ADHD patients. Low serum ferritin has been found both in ADHD patients as well as in patients with narcolepsy.

Materials & methods

We enrolled 26 participants (14 ADHD patients and 12 controls). They answered several questionnaires, and blood samples were obtained from 20 participants. We had clear exclusion criteria.

Results

Using the Ullanlinna Narcolepsy Scale (UNS), we identified three possible narcolepsy patients within the ADHD group and no suspects in the control group. There was a statistically significant negative correlation between serum iron levels and ADHD symptom severity. No correlation was found measuring serum ferritin levels.

Conclusions

Narcolepsy may be more common within ADHD patients than in the general population. Some of these patients could benefit from a change in medication. Low serum iron and ferritin levels could be relevant in ADHD pathophysiology. This requires further exploratory research.

## Introduction

Epidemiology of attention-deficit/hyperactivity disorder (ADHD)

ADHD is a psychiatric disorder characterized by inattention, hyperactivity, and impulsiveness, with an estimated worldwide prevalence of 7.2%, according to a meta-analysis [1]. The diagnostic criteria divide the patients into either inattentive, hyperactive-impulsive, or a combined presentation. Interestingly, ADHD in children and adolescents is much more common in the young male population (the ratio is 4:1) [2]. ADHD persists into adulthood in about 60% of all cases and is therefore a very common disease even among adults [3].

Pathophysiology of ADHD

The pathophysiology of ADHD is not yet fully understood, but it has been proposed that problems could occur because of hypoactive monoaminergic neurons, which project to the prefrontal cortex. This hypothesis has been founded on the phenomenon where the prefrontal cortical functions of ADHD patients are briefly hypoactive [4]. Astrocytes, energy deficiency, and the hypothesized lactate shuttle may be involved too. Glucose is taken up from the blood by astrocytes. As this undergoes glycolysis, pyruvate and adenosine triphosphate (ATP) are produced. Further, pyruvate can either be converted to glycogen for storage or be metabolized to lactate. The lactate shuttle is now able to transfer lactate from astrocytes to neurons, where it is used as energy. Catecholamines (dopamine and noradrenaline) bind to their respective receptors on astrocytes inducing glycogenolysis [5]. This results in elevated glucose concentrations and lactate production. Therefore, reduced catecholamine levels could cause lower lactate concentrations and less energy in the neurons. This lack of energy could be the reason behind the reduced prefrontal cortical function [4].

Comorbidities in ADHD

ADHD patients are at greater risk for several psychiatric disorders such as depression, bipolar disorder, and anxiety disorders [6]. Overlapping and distinctive features between these disorders are, however, notable [6]. According to Katzman et al., substance use disorder and personality disorders are more common among ADHD patients than in the general population. Up to 44% of all ADHD children suffer from restless legs syndrome (RLS), and 26% of RLS patients are reported suffering from ADHD or at least severe ADHD symptoms [7]. Iron deficiency in children with ADHD and lower ferritin levels have been associated with the severity of RLS symptoms [8-9]. In a meta-analysis, it was found that peripheral serum ferritin levels were lower for children with ADHD than for controls but serum iron and transferrin levels did not differ [10]. Percinel et al. found no difference in iron parameters between ADHD patients and controls in children [11]. Hyperactivity scores, on the other hand, negatively correlated with serum ferritin levels. The prevalence of ADHD in women with iron deficiency anemia could be higher than in the general population [12].

Pharmacological treatment in ADHD

Stimulants (methylphenidate and amphetamines) are first-line pharmacological agents and the most commonly used in ADHD. Methylphenidate appears to be the most efficient treatment [13]. Atomoxetine is also commonly used and is superior to a placebo [13]. Another promising non-stimulant is modafinil. According to a double-blind study, it appears to improve ADHD significantly as compared to placebo [14]. Modafinil is the most commonly used medication in narcolepsy, and it is also used as an off-label medication in ADHD.

Narcolepsy

Narcolepsy is a chronic neurological condition. It is characterized by two main symptoms, excessive daytime sleepiness (EDS) and cataplexy. The reported global mean prevalence is approximately 30 per 10,000 [15]. Other symptoms are, for example, sleep paralysis and hypnagogic hallucinations. Narcolepsy was first divided into two subtypes: narcolepsy Type 1 (with cataplexy) and narcolepsy Type 2 (without cataplexy) by the International Classification of Sleep Disorders (ICSD). This subdivision has now been replaced by the ICSD-3, according to which Type 1 narcolepsy (NT1) is with hypocretin (also called orexin) deficiency and Type 2 narcolepsy (NT2) is without hypocretin deficiency, e.g. the absence of cataplexy. The HLA-DQB1*06:02 allele has been associated with narcolepsy. According to a population-stratified analysis, HLA-DQB1*06:02 increased the risk for both NT1 and NT2, but the odds ratio was significantly higher for NT1 than NT2 (24.1 vs. 3.9) [16]. Methylphenidate has been used in the treatment of narcolepsy but, nowadays, modafinil is more commonly used [17]. Modafinil is used particularly for treating EDS, atomoxetine and venlafaxine are first-line medications for cataplexy, but pitolisant could be both safe and efficient [17-18].

Relationship between ADHD and narcolepsy

Three out of 15 ADHD children showed narcolepsy-like symptoms during their sleep [19]. Although researchers at this time do not know if the children suffered from narcolepsy, this high percentage (20%) serves as an indicator for possible comorbidity. Causality is unclear because sleep disorders can induce ADHD-like symptoms, which could be due to excessive daytime sleepiness [20]. Narcoleptic patients are more prone to ADHD than the general population. As much as 30% of all patients suffering from narcolepsy without cataplexy and 15% of narcoleptics with cataplexy had symptoms of ADHD [21]. In a Swedish study, 25% of all narcolepsy patients had ADHD [22]. The same study revealed no correlation between hypocretin levels and ADHD symptoms. Narcoleptics have a higher prevalence of major depression than the general population [22]. Also, temper tantrums are very common in narcolepsy [22].

Aims

ADHD patients often suffer from daytime sleepiness; in addition, some of them are also using medications as in narcolepsy (e.g., methylphenidate), and it is possible that at least mild forms of narcolepsy remain masked. Our aim was to clarify the prevalence of narcolepsy symptoms among adult ADHD patients as compared to controls of the same age and gender. This included defining the appearance of daytime sleepiness and cataplectic symptoms. We also wanted to evaluate the association of low serum ferritin and iron levels to ADHD and symptoms of narcolepsy.

## Materials and methods

Subjects and clinical assessments

Fourteen adult ADHD patients (9 women and 5 men; age range 23 - 48 years) and 12 age-matched (± 10 years) healthy controls (8 women and 4 men) were included. The information was gathered during the timeframe of March 2015 to October 2019. Exclusion criteria for controls included a prior diagnosis of ADHD or narcolepsy. The ADHD patients had either been previously diagnosed by an experienced psychiatrist or were recruited from the Finnish union for ADHD patients. The DSM-5 diagnostic criteria of the American Psychiatric Association for ADHD were used [23]. The controls were workers from different healthcare organizations and other healthy volunteers. We excluded all patients and controls who had been diagnosed with a psychotic disease or had been prescribed an antipsychotic drug. Neither did we include patients who had received treatment in the form of tricyclic antidepressants, selective serotonin reuptake inhibitors (SSRIs), clonidine, or atomoxetine since these could have reduced cataplectic symptoms. We also excluded patients and controls that scored > 7 on the Alcohol Use Disorder Identification Test (AUDIT), as well as subjects that had misused intoxicating substances such as methylphenidate or modafinil. Patients and healthy controls matched in terms of years of education (± five years). All patients had a Caucasian origin. All participants had been informed on what they were to undergo, and written informed consent was obtained in all cases.

Questionnaires

We used a structured questionnaire based on the Basic Nordic Sleep Questionnaire (BNSQ) to chart the participants for narcoleptic symptoms, other sleep disorders, ADHD severity, and general well-being. For the mapping of the narcoleptic symptoms, the Ullanlinna Narcolepsy Scale (UNS) was also used [24]. The questionnaire also included the Epworth Sleepiness Scale (ESS), World Health Organisation-Five Well-Being Index (WHO-5), and questions on the sleep-wake rhythm and other sleep disorders. The questionnaire can be obtained by request from the authors free of charge. All participants responded to self-administered questionnaires. We mapped ADHD symptoms using the Wender Utah Rating Scale (WURS) and the Adult ADHD Self-Report Scale (ASRS) version 1.1. 10 ADHD patients and 10 controls answered all questionnaires.

Other assessments

The clinical assessments included taking of the medical history and computing the Body Mass Index (BMI; kg/m^2^) was computed from weight and height. Serum samples were analyzed in an accredited laboratory of the University of Helsinki.

Statistical methods and ethical aspects

Statistical computations were done using Stata 15.1 (StataCorp, Texas). The normality of the distributions was tested by the Shapiro-Wilk test. Parametric or non-parametric tests were used according to the distribution. Means and 95% confidence intervals (CI) were computed. For normally distributed variables Student’s t-test and linear regression were used. Fisher’s exact test was used in cross-tabulations. We used two-sided P-values with a limit of significance of P <0.05. The ethical approval was given by the Helsinki and Uusimaa District Ethical Committee.

## Results

The age among the participants was normally distributed (Shapiro-Wilk normality test, P-value = 0.18342). The gender (8 women and 6 men among patients vs. 9 women and 3 men among controls) of the participants did not statistically differ (Fisher’s exact = 0.429) and the age did not differ among groups (means: 38.2 vs. 34.6, P = 0.3084) (Table 1).

**Table 1:** Differences between ADHD and control subjects Statistical difference (P-value) was computed according to the distribution by Fisher’s exact test (categorical variables), Student’s t-test (age, ASRS total sum, and serum ferritin), or Mann-Whitney U-test (serum iron). ADHD: attention-deficit/hyperactivity disorder; ASRS: adult ADHD self-report scale

	ADHD Occurrence (%) or Median; Mean (95% CI)	Controls Occurrence (%) or Median; Mean (95% CI)	P-Value
Gender: women/men	57% / 43%	75% / 25%	0.429
Age (y)	39.2 y; 38.2 (33 to 42)	34.6 y; 34.6 (28 to 41)	0.308
ASRS (part A) ≥ 4	70 %	0 %	0.003
ASRS (total sum)	64; 62.8 (56.7 to 68.9)	34.5; 37 (31.3 to 42.7)	<0.001
Serum iron (umol/L)	12.6 (9.4 to 15.8)	17.9 (13.6 to 22.2)	0.026
Serum ferritin (ug/L)	80.6 (26.6 to 134.6)	86 (47.6 to 124.4)	0.851

Narcoleptic symptoms

With UNS, we managed to compare both groups for EDS and cataplexy. In the clinic, a cut-off point of ≥ 13 is used for screening for narcolepsy. Using this, three out of 14 patients, whereas no controls were screened positive for narcolepsy. However, this was not statistically significant (Fisher’s exact = 0.225). A two-sample T-test revealed no difference between the groups (means: 7 vs. 4.4, P = 0.1247). When only counting the points for cataplexy in UNS, we used a cut-off point of ≥ 3 for addressing cataplexy. This point was chosen due to it being the best point for separating between if the symptoms are felt on a weekly-monthly basis or not. Using this, five out of 14 ADHD patients screened positive for cataplectic symptoms, whereas no controls did. This difference was statistically significant (Fisher’s exact P = 0.042). For EDS, five out of 14 screened positive and one out of 12 controls also did. This was not statistically significant (Fisher’s exact = 0.170). Using ESS, a two-sample t-test revealed no difference between the groups (means: 5.6 vs. 3.8, P = 0.1907).

Comparing the groups' ADHD symptoms and well-being

The prevalence of WURS scores greater than 44 (likely ADHD) was nine out of nine in the ADHD group and three out of 10 in the control group. The lowest WURS score in the ADHD group was higher than the highest score from the controls, indicating a strong difference between groups. Using the ASRS questionnaire as a diagnostic tool, where on getting a score of ≥ 4 in part A, none of the controls had adulthood ADHD, whereas seven out of 10 of the ADHD group qualified. A two-sample t-test, comparing WHO-5 scores among groups, revealed a statistically significant difference in favor of controls (means: 48.6 vs. 64.7, P = 0.0322). Comparing patients with UNS ≥ 13 with the others for WHO-5 scores revealed no difference between groups (means: 62 vs. 58.1, P = 0.7899).

Iron levels and ADHD

**Figure 1:**
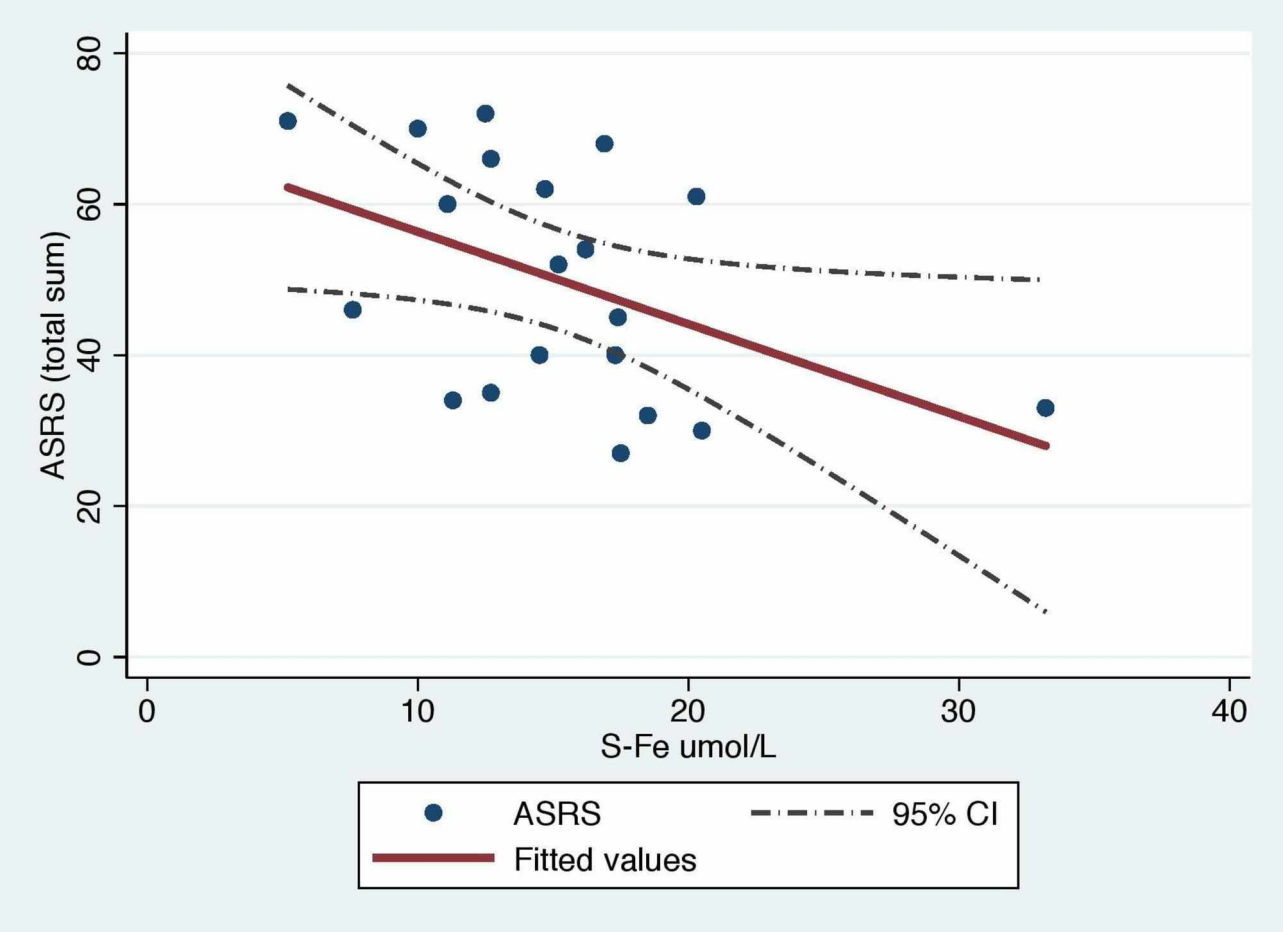
Linear regression with the ASRS scores and serum iron levels as variables ASRS: adult attention-deficit/hyperactivity disorder (ADHD) self-report scale

We used the whole ASRS questionnaire to get a score for the severity. This was done by adding up all 18 questions. The serum iron and ASRS score negatively correlated (cor = -0.56, calculated with Spearman’s rank correlation coefficient formula), and a linear regression revealed the statistical significance of this negative correlation (p-value = 0.041). This correlation is illustrated in Figure 1. The serum iron score was not normally distributed (P-value = 0.047 in a Shapiro-Wilk normality test), and, therefore, we used Spearman’s test for calculating correlations. Serum iron levels were higher for controls than for ADHD patients (means: 12.6 vs 17.9, P = 0.0384) but regarding serum ferritin levels, we identified no difference (means: 80.6 vs 86, P = 0.8511). Blood hemoglobin levels did not differ (means: 141.3 vs 141.9, p-value = 0.89) among groups.

## Discussion

As far as we know, no studies comparing adult ADHD patients with healthy controls, regarding narcoleptic symptoms, has been made before. UNS revealed three possible narcolepsy patients in the ADHD group. And this is, although not statistically relevant, at least clinically relevant. The pharmacological treatment of EDS experienced in narcolepsy resembles the pharmacological treatment in ADHD, but there are differences. One could hypothesize whether these three patients are receiving the wrong treatment. Could the patients possibly be doing better if receiving modafinil instead of methylphenidate, as is the case now? In our study, only two of the possible narcoleptics answered the WHO-5, making a statistical analysis comparing these persons to others unreliable.

We strongly believe that a bigger cohort could reveal a statistical difference between the groups. If the prevalence of narcolepsy would be as big as in this study (≈21%), it would mean that the global prevalence of narcoleptic symptoms would be higher than what is assumed at the present. A study revealed a mean UNS score of 22.0 within NT1 patients and 13.7 in NT2 [24]. The UNS scores for the NT1 patients were higher in that study than for any patients in the present study. We cannot make final inferences regarding this issue. It is also possible that our three patients with UNS ≥ 13 could have narcolepsy Type 2.

ADHD patients have more cataplectic symptoms than controls. What we do not know is if the patients affected experience these symptoms as handicapping. One could also speculate whether these patients could benefit from a cataplexy-reducing agent. Considering atomoxetine, which is a second-line agent for ADHD but first-line treatment for cataplexy, the question is: whether these patients would benefit from an atomoxetine medication, possibly allowing them to stop their stimulant medication.

We did not find any differences between the groups regarding excessive daytime sleepiness either from the questions regarding EDS in UNS nor from ESS. It has to be kept in mind that all ADHD patients use stimulants on a daily basis, which reduces EDS symptoms. It would be very interesting to see a study comparing adult ADHD patients not receiving stimulants with healthy controls regarding this subject.

We found a statistically significant negative correlation between serum iron levels and ADHD symptom severity, as well as a statistically significant difference between the groups regarding serum iron levels. Serum ferritin levels have been believed to estimate the bodies' iron reservoir in a better manner than serum iron, but we found no statistical correlation between serum ferritin levels and ADHD symptom severity. With a bigger sample, we would get more clear estimates on the possible correlations and, therefore, the relevance of these markers.

Unfortunately, our study has several weaknesses. The sample size is small. Finding participants was even more difficult than we expected. We are aiming to find more subjects, which is, however, even more difficult during the present time (COVID-19). Due to the present situation, obtaining blood samples and actigraphic measures from all participants was not timely. The samples taken were obtained in 2015-2016. This led to the fact that a part of the participants didn’t have results on some variables. We didn’t measure hypocretin levels in the cerebrospinal fluid (CSF), which is a more specific diagnostic tool for narcolepsy Type 1 than UNS. With hypocretin levels, we could estimate the prevalence more precisely. However, NT2 patients have normal CSF-hypocretin levels. Therefore we cannot exclude the possibility that some of our patients may have had low CSF-hypocretin levels.

## Conclusions

Our study shows that narcolepsy may be more common in subjects with ADHD than in subjects without ADHD. If this is the case, one could hypothesize that these patients could benefit from a change in medication. Serum ferritin levels are widely used clinically for various purposes. Serum-free iron levels, however, are not as commonly measured or used. Serum iron levels correlated negatively with the ASRS score. More studies are needed in this regard. At the present time, we do not know whether ADHD patients or a sub-group of ADHD patients could benefit from iron supplementation. We also need randomized controlled studies on the use of iron supplementation in patients with narcolepsy.
